# Influence of chemical conditioning and micromechanical roughening on bond strength to zirconia ceramic

**DOI:** 10.1186/s12903-025-07628-1

**Published:** 2026-01-27

**Authors:** Ahmed Abo Khalil, Mohamed Ellayeh, Ahmed Attia

**Affiliations:** Department of Fixed Prosthodontics, Faculty of Dentistry, Mansoura University, El Gomhouria St, Mansoura, Dakahlia Governorate Egypt

**Keywords:** Zirconia, Chemical conditioning, Surface treatment, Resin cement

## Abstract

**Background:**

The objective of the present in-vitro study was to evaluate the effect of different surface treatment methods with and without chemical conditioning on bonding to zirconia ceramic.

**Methods:**

A total of 64 disc-shaped specimens (3 mm in thickness and 8 mm in diameter) were fabricated and divided according to surface treatment into four main groups (*n* = 16): as milled by CAD CAM with no further treatment (control group) (CNT); airborne-particle abrasion with AL_2_O_3,_ (APA); etched by ammonium hydrogen difluoride (ABF), etched by a zirconia etching system (ZES). Each main group was divided into two subgroups (*n* = 8) according to use of primer: Primer application (P) or no primer application (NP) to form a total of 8 test groups as follow; CNT-NP, CNT-P, APA-NP, APA-P, ABF-NP, ABF-P, ZES-NP and ZES-P. Transparent plastic tubes were filled with composite resin and bonded to zirconia discs using adhesive resin cement. All specimens were artificially aged. Hydrolytic ageing (5 months, 37 °C) and thermalcycling (x5000, 5–55 °C) were applied. Tensile bond strength (TBS) was recorded in MPa using a universal testing machine. Statistical analysis was performed using 2-way ANOVA to assess the effects of surface treatment and primer application on bond strength followed by serial 1-way (ANOVA)s test and post hoc (LSD) test for pairwise comparisons.

**Results:**

Chemical conditioning by primer application showed a significant effect on TBS measurements (*P* = 0.014), whereas micro-mechanical surface treatment did not show a significant difference (*P* = 0.47), and the interaction between the two factors was also not significant (*P* = 0.14). Group APA-P showed the highest mean TBS (7 ± 2.8, *P* < 0.05), while group CNT-NP showed the lowest mean TBS (3.4 ± 0.7, *P* > 0.05).

**Conclusion:**

Primer application significantly improved bond strength to zirconia ceramic regardless of the different techniques used for micromechanical surface treatment.

## Background

 Recently, partially stabilized zirconia ceramics are becoming more common in dental practice due to its excellent physical, highly aesthetic, and biocompatible properties [1, 2]. The new software and technologies involved in dental restorations fabrication paved the way for utilizing zirconia more frequently for prosthetic restorations [3]. The cementation and bonding processes of ceramic restorations to the tooth structure determine their success. Conventional cements can be used with zirconia restorations. While, resin cements can be used in cases that require higher retention offering better marginal adaptation and improved longevity of the ceramic restoration [4, 5]. 

Bonding between zirconia ceramics and resin cements remain controversial, as application of common adhesive cementation techniques, which are acid etching then silanization of ceramic surface, are not able to produce durable bond strength to oxide ceramics. Micromechanical bonding is required to attain an efficient and long lasting adhesion between resin cement and zirconia [6]. Different surface treatment methods were used to modify zirconia surfaces to evaluate their impact on adhesion to resin cement [5, 7, 8]. The most popular technique for zirconia surface treatment is airborne-particle abrasion [9, 10]. Numerous studies revealed that the abrasion technique has a role in boosting the strength of the adhesion between resin cements and zirconia restorations. However, this might induce zirconia surface crystalline structure to transform from tetragonal phase to monoclinic phase which will result in micro-cracks formation which in turn would reduce the zirconia stability [11, 12]. Furthermore, the size of alumina particles, the distance and pressure of air application, and the uniformity of surface abrasion all influence the airborne-particle abrasion process [10, 13, 14]. This effect becomes more pronounced when airborne-particle abrasion is performed at a shorter application distance or with larger abrasive particles, which causes microcrack formation on the zirconia surface. These drawbacks lowers the long-term mechanical features of the ceramic and complicates the application of uniform surface treatment in dental clinics [15]. Recently, many studies have focused on modifying zirconia surfaces using different methods such as acid etching. Various studies examined the use of chemical etching through different methods to enhance adhesive strength and eliminate the limitations associated with the airborne-particle abrasion technique [16–21]. 

Chemical bond formation and micromechanical interlocking are important factors for achieving durable long lasting and strong bond between zirconia ceramics and resin cement [22]. Multiple primers containing acidic groups such as 10-methacryloxydecyldihydrogen phosphate (MDP) were developed to enable the chemical bonding to zirconia [5, 23–26]. Universal primers containing MDP monomer and 10-MDP resin cement can chemically adhere to the metal oxides on the surface of the zirconia ceramics by forces of van der Waals or hydrogen bonds at the interface between the resin and the zirconia surface, yielding an initial high bond strength with zirconia [24, 27]. A study that evaluated the impact of chemical conditioning previously revealed that using primer has more importance in the bonding process than the cement type [28]. 

Despite the extensive research on zirconia bonding, there is still no consensus regarding the optimal surface treatment protocol that ensures reliable and durable adhesion without compromising the structural integrity of zirconia. Each method presents advantages and drawbacks, particularly concerning surface damage, phase transformation, and bond longevity. Therefore, further investigation is essential to compare newly developed surface treatment systems, such as the zirconia etchant cloud system, with the conventional airborne-particle abrasion technique. This study aims to provide deeper insight into how these micromechanical surface treatments, with or without chemical conditioning by primer application, influence the bonding performance of zirconia ceramics ultimately guiding clinicians toward more predictable and effective adhesive protocols.

Therefore the purpose of this invitro study was to evaluate the effect of three micromechanical surface treatment methods: airborne-particle abrasion, etching by ammonium hydrogen difluoride and zirconia etchant cloud system without or with chemical conditioning on bonding to zirconia ceramics. The null hypothesis of the study was that micromechanical surface treatment and chemical conditioning would not have effect on the tensile bond strength (TBS) to zirconia.

## Materials and methods

Details of the materials used in this study are shown in (Table 1).

**Table 1:** Materials used in the study

Material type	Product name	composition	Manufacturer	Lot number
Zirconia ceramic blanks for CAD/CAM	IPS e.max-ZirCAD	(in % by weight) ZrO_2_: 87–95 Y_2_O_3_: 4–6 HfO_2_: 1–5 Al_2_O_3_: 0.1-1	Ivoclar Vivadent, Schaan, Liechtenstein	X46187
110 μm Al_2_O_3_ for airborne-particle abrasion	Sahara	99.7% aluminum oxide	Dentify GmbH, Engen, Germany.	1366
Ammonium hydrogen difluoride (NH_4_HF_2_)	Ammonium bi-fluoride	Assay (acidimetric): minimum 98% Maximum limits of impurities: Chloride: 0.002% Nonvolatile matter: 0.05% silica: 0.5% iron:0.005% lead: 0.01% sulphate: 0.05%	Qualikems fine chem pvt, Ltd. Nandesari, Vadodara, Gujarat. India	A027012
Zirconia etching system	Zirconia etchant cloud system	> 10% hydrofluoric acid > 2% thickening agent Ferric chloride, methanol, HF neutralizer gel.	Mdeifive, Korea.	116,112 K
Light cure composite resin	Alpha Dent	Radiopaque fillers, Bis-GMA, Comonomer dilutents, accelerator, Stabilizer, photoInitiator.	Dental Technologies, USA.	G164HS
Universal primer	Monobond N	Butanol, dihydrogen trifluoride, tetrabutylammonium, methacrylated phosphoric acid ester and Bis(triethoxysilyl)ethane	Ivoclar Vivadent	Z03CXK
Adhesive resin dental cement	Multilink N	HEMA, Dimethacrylate, ytterbium, spherical mixed oxides, barium glass, tri-fluoride.	Ivoclar Vivadent	Z053Z4

## Methods

### Power analysis

Power analysis and calculation of the required sample size were performed using G*Power 3 software and revealed that power range of the experiment is 52–60. The total sample size of the experiment was 64 [4, 5, 8, 14]. 

###  Zirconia discs preparation

Zirconia ceramic discs (*n* = 64) were dry milled from monolithic zirconia ceramic blanks (IPS Emax-ZirCAD, Ivoclar-Vivadent, FL) with (8 mm diameter and 3 mm thickness) [4, 5, 8, 14] using CAD/CAM milling machine (Roland DWX-52D Hamamatsu, Japan). Sintering of zirconia discs was carried out according to the manufacturer instructions. The discs were divided into 4 main groups (*n* = 16) according to the type of micromechanical surface treatment technique. Each main group was divided into two sub-groups (*n* = 8) based on chemical conditioning a total of 8 test groups were resulted: CNT-NP: as milled no primer application, CNT-P: as milled plus primer application, APA-NP: airborne-particle abrasion no primer application, APA-P: airborne-particle abrasion plus primer application, ABF-NP: etching with NH_4_HF_2_ no primer application, ABF-P: etching with NH_4_HF_2_ plus primer application, ZES-NP: etchant cloud system no primer application and ZES-P: etchant cloud system plus primer application (Table 2).

**Table 2:** Micromechanical roughening and chemical conditioning of test groups

Group	Abbreviation	Material	Surface traetment	Manufacturer	Application parameters
as milled no primer application	CNT-NP		No micro-mechanical surface treatment		
as milled plus primer application	CNT-P				
airborne-particle abrasion no primer	APA-NP	Al_2_O_3_ particles 110 μm	airborne-particle abrasion	Dentify GmbH, Scheffelstraße, Engen, Germany.	Pressure of 2 bars for 15 s with 10 mm distance perpendicular to discs bonding surfaces using a sandblaster (Renfert, Germany).
airborne-particle abrasion plus primer application	APA-P				
etching with NH_4_HF_2_ no primer application	ABF-NP	Viscous slurries of NH_4_HF_2_ were spread on the bonding surfaces of zirconia discs.	Etching by Ammonium hydrogen difluoride	Qualikems fine chem pvt, Ltd. Nandesari, Vadodara, Gujarat.	The discs were etched by NH_4_HF_2_ for 10 min at 170 °C
etching with NH_4_HF_2_ plus primer application	ABF-P				
etchant cloud system no primer application	ZES-NP	HF acid gel in the form of Zirconia etchant cloud system	Etching by HF acid	Mdeifive, Korea.	Etching done in a safe shell for 10 min.
etchant cloud system plus primer application	ZES-P				

**Table 3:** Showing Mean ± SD (TBS) of all test groups in (MPa) and results of post-hoc LSD test

	CNT-NP	CNT-*P*	APA-NP	APA-*P*	ABF-NP	ABF-*P*	ZES-NP	ZES-*P*
Mean ±SD	3.4 ± 0.7	6.4 ± 1.4	4.2 ± 2.6	7 ± 2.8	5.8 ± 3.4	6.4 ± 1.9	6.3 ± 1.6	6 ± 2.9
CNT-NP		0.023 *	0.531	0.005 *	0.076	0.020 *	0.026 *	0.056
CNT-P			0.069	0.522	0.616	0.962	0.958	0.729
APA-NP				0.016 *	0.204	0.063	0.077	0.155
APA-P					0.265	0.553	0.489	0.336
ABF-NP						0.584	0.652	0.879
ABF-P							0.920	0.695
ZES-NP								0.768
ZES-P								

*CNT-NP* as milled no primer, *CNT-P* as milled plus primer, *APA-NP* airborne-particle abrasion no primer, *APA-P* airborne-particle abrasion plus primer, *ABF-NP* etching with NH4HF2 no primer, *ABF-P* etching with NH4HF2 plus primer, *ZES-NP* etchant cloud system no primer application, *ZES-P* etchant cloud system plus primer application

*Indicate statistically significant differences at (*p* < 0.05)

#### As milled

Sixteen zirconia discs were used for bonding as milled without further micromechanical treatmen.

#### Airborne-particle abrasion

Al_2_O_3_ particles measuring 110 μm (99.7% aluminum oxide Dentify GmbH, Engen, Germany) were used for airborne-particle abrasion of the bonding surfaces of 16 zirconia discs under a pressure of 2 bars for 15 s with 10 mm distance perpendicular to discs bonding surfaces using a sandblaster (Renfert, Hilzingen, Germany) [10]. 

#### Ammonium hydrogen difluoride 

Bonding surfaces of 16 zirconia discs were etched by NH_4_HF_2_ (Qualikems fine chem pvt, Ltd., Vadodara, Gujarat, India) as follow: Viscous slurries of the material were formed by mixing 4.2 mg/mL NH₄HF₂ powder with distilled water, the formed slurries were subsequently applied to the bonding surfaces of zirconia discs. Heating of the discs was carried out in a furnace. The discs were etched by NH_4_HF_2_ for 10 min at 170 °C [19, 20].

#### Etchant cloud system (Medifive, Incheon, Republic of Korea)

Bonding surfaces of 16 zirconia discs were etched by Hydrofluoric acid (HF) gel according to the manufacturer instructions as follow: The bonding surfaces of the discs were covered by HF acid gel and then placed in the safe shell that was closed after immersing the pack into water at the bottom and the neutralizer gel was applied to the top opening. The reaction was carried out for 10 min then zirconia discs were collected [16]. Finally all specimens were collected and rinsed in water then cleaned in ultrasonic bath at 99% alcohol for 3 min and dried [5, 14]. A thin coat of universal primer (Monobond N, Ivoclar Vivadent, FL) was then applied to 8 specimens of each main group with a micro-brush (Ivoclar Vivadent, FL) and left to react for 60 s. Subsequently, excess primer was dispersed with an air stream according to the manufacturer instructions.

#### Bonding of specimens

Bonding procedures were conducted according to other published studies [5, 8, 14]. Plexiglas tubes (Rohn, Darmstadt, Germany) with standardized diameter (3.2 mm) were filled with light polymerizing restorative composite resin (Alpha Dent, Dental Technologies, IL, USA) and bonded to the conditioned intaglio surfaces of zirconia discs. Adhesive resin cement in dual-polymerizing mode (Multilink N, Ivoclar Vivadent, FL) was used for bonding the plastic tubes to the pretreated ceramic discs in accordance with the manufacturer´s recommendations. Excess resin cement was removed using disposable microbrushes (Ivoclar Vivadent, FL). Light polymerization was done at four directions with 2 mm distance using light polymerizing unit (DB686 Nano light cure unit, Coxo, Foshan, China) for 40s with an output of 650 mW/cm^2^. A custom-designed device was used to apply the constant load of 5 kg for 10 min on the assembly [4, 5, 8, 14]. After cementation, the bonded specimens were left for one hour at room temperature to ensure complete polymerization of the dual-cured resin cement and to relieve initial polymerization stresses. Subsequently, all specimens were stored in distilled water at 37 °C for five months to simulate hydrolytic ageing and prolonged exposure to the humid oral environment before testing [4, 5, 8, 14]. Additionally, thermal cycling was performed to further simulate intraoral temperature fluctuations. Specimens were subjected to thermalcycling for 5000 cycles using thermalcycling device (Julabo FT200, Seelbach, Germany). Each cycle consisted of 1 min in 5 °C cold bath and 1 min in 55 °C hot bath with 30 s dwell time then dried before testing. A universal testing machine (Instron 3345, Norwood, MA, USA) equipped with a 5 kN load cell was used to record the TBS at a cross-head speed of 2 mm/min [5, 8] using a chain loop alignment which provided a moment free axial application. The necessary load for de-bonding was recorded in Newtons (N) and divided by the surface area and expressed in MPa.

$$\tau=P/\pi r^2$$

where ; τ = TBS (MPa, P = load at failure(N).

π = 3.14 and r = radius of resin disc(mm).

### Statistical analysis

Statistical analysis of data was conducted using Social Package for Statistical Sciences (SPSS) software version 26.0. (SPSS Inc. Chicago, Ill, USA) and G*Power 3. Statistical analysis was performed with two-way ANOVA test at two factors of the study followed by serial one-way (ANOVA)s test for each factor and post hoc Least Significant Difference (LSD) for pairwise compression between each two test groups.

## Results

### Normality test

The normality test was performed using Kolmogorov-Smirnov test. Data were found to be normally distributed therefore parametric methods were used for statistical analyses.

### Statistical analysis

Two-way ANOVA demonstrated that chemical conditioning by primer application showed a statistically significant effect (*P* = 0.014). On the other hand micromechanical surface treatment was not significant (*P* = 0.46). Also the interaction between the two factors was not significant (*P* = 0.14). To study the effect of each factor, one-way ANOVA tests confirmed that only chemical conditioning with primer had a significant effect (*P* = 0.018), while micromechanical surface treatment factor was not significant (*P* = 0.65). Means ± SD TBS values in (MPa) for all test groups along with pairwise comparisons using the post hoc (LSD) test at significance level (*P* ≤ 0.05) are presented in (Table 3).

CNT-NP (control) group exhibited the lowest TBS (3.4 ± 0.7) among all test groups, while APA-P group showed the highest TBS (7 ± 2.8).

**Fig. 1:**
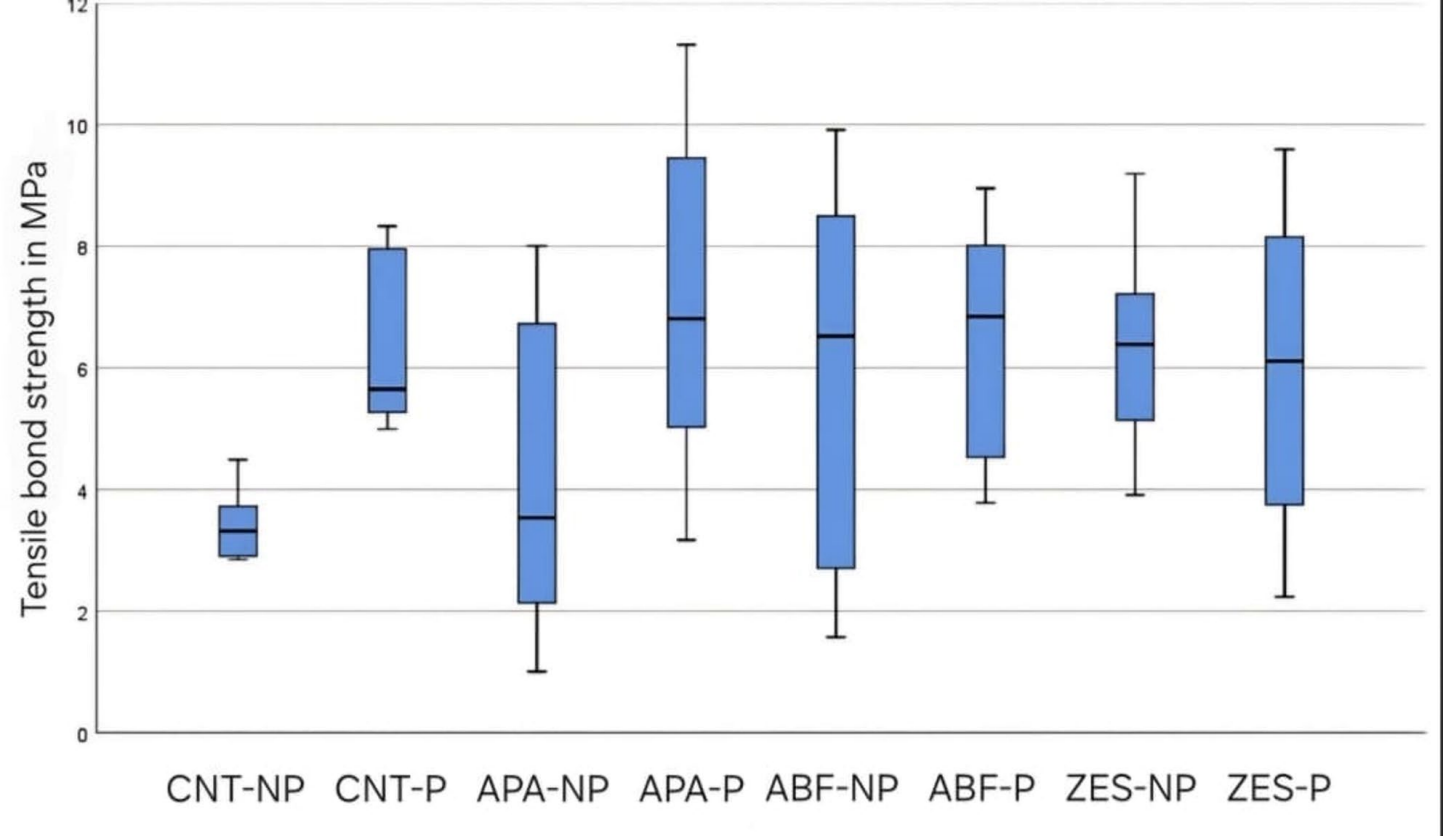
Boxplot showing means TBS of test groups

When comparing test groups without primer application, a significant difference was found between CNT-NP and ZES-NP (*P* = 0.026), while other pairwise comparisons among CNT-NP, APA-NP, ABF-NP, and ZES-NP were not significant (*P* > 0.05). Considering test groups with primer application, no significant differences were observed among CNT-P, APA-P, ABF-P or ZES-P test groups (*P* < 0.05). When comparing micro-mechanical surface treatment with and without primer application: There were statistically significant differences between the following test groups; CNT-P (6.4 ± 1.4) - CNT-NP (3.4 ± 0.7), (*P* = 0.023). APA-P (7 ± 2.8), APA-NP (4.2 ± 2.6), (*P* = 0.016). However there were no statistically significant differences (*P* < 0.05) between the other test groups; ABF-P (6.4 ± 1.9) - ABF-NP (5.8 ± 3.4), ZES-NP (6.3 ± 1.6) - ZES-P (6 ± 2.9). Groups APA-P, ABF-P, and ZES-NP showed significantly higher mean TBS compared to group CNT-NP, (*P* < 0.05) but did not differ significantly from group CNT-P, (*P* < 0.05). Box-Plot was used to confirm the results of the pair-wise comparison (Fig. 1).

### Failure pattern analysis

**Fig. 2:**
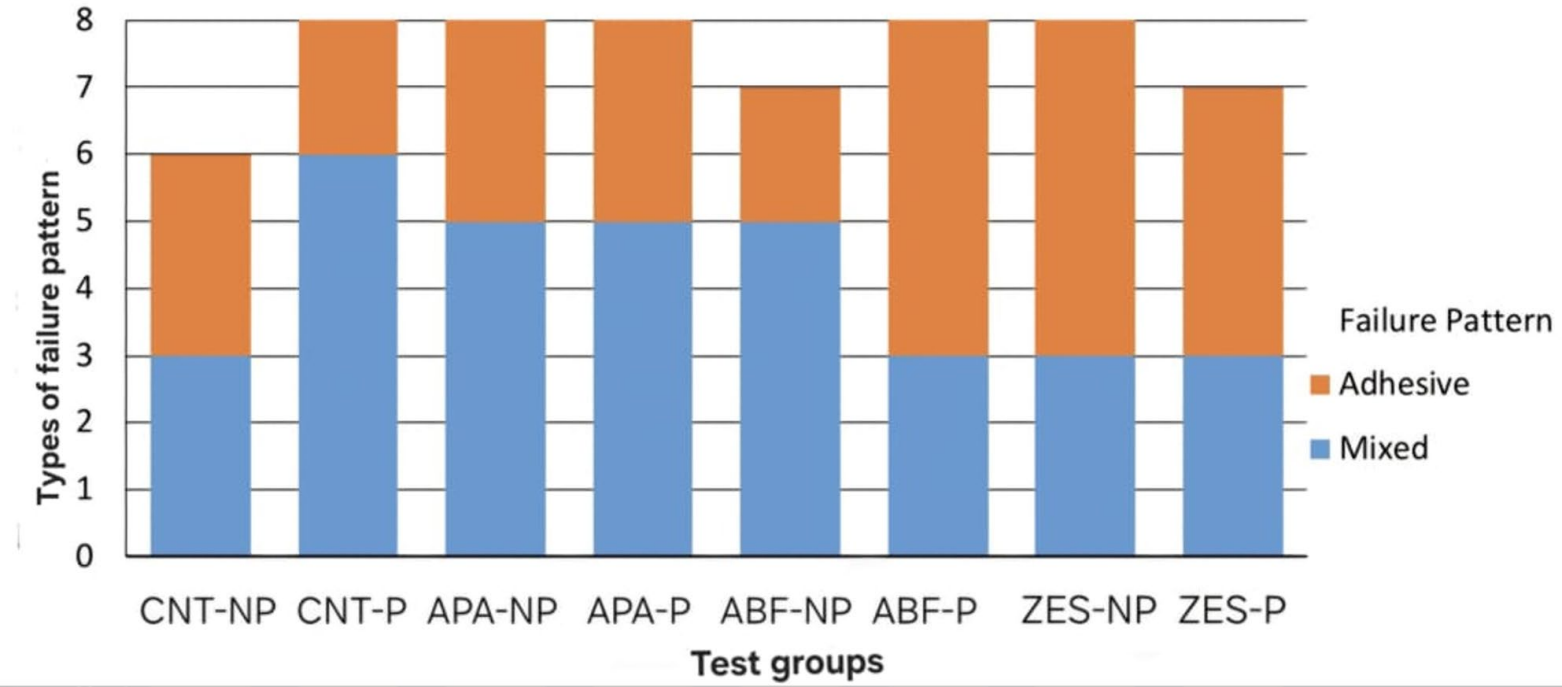
Bar-chart of failure pattern among test groups

**Fig. 3:**
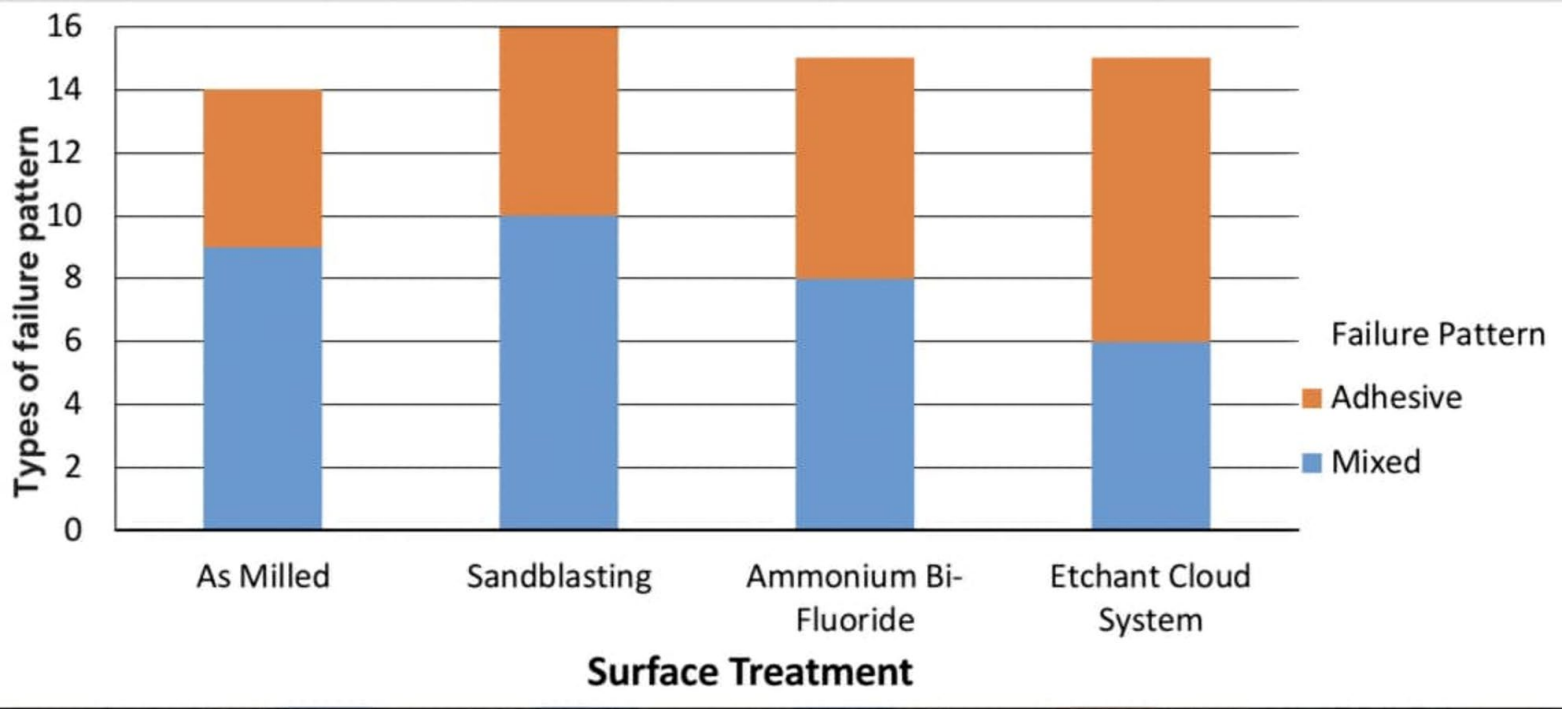
Bar-chart of failure pattern considering micromechanical surface treatment

**Fig. 4:**
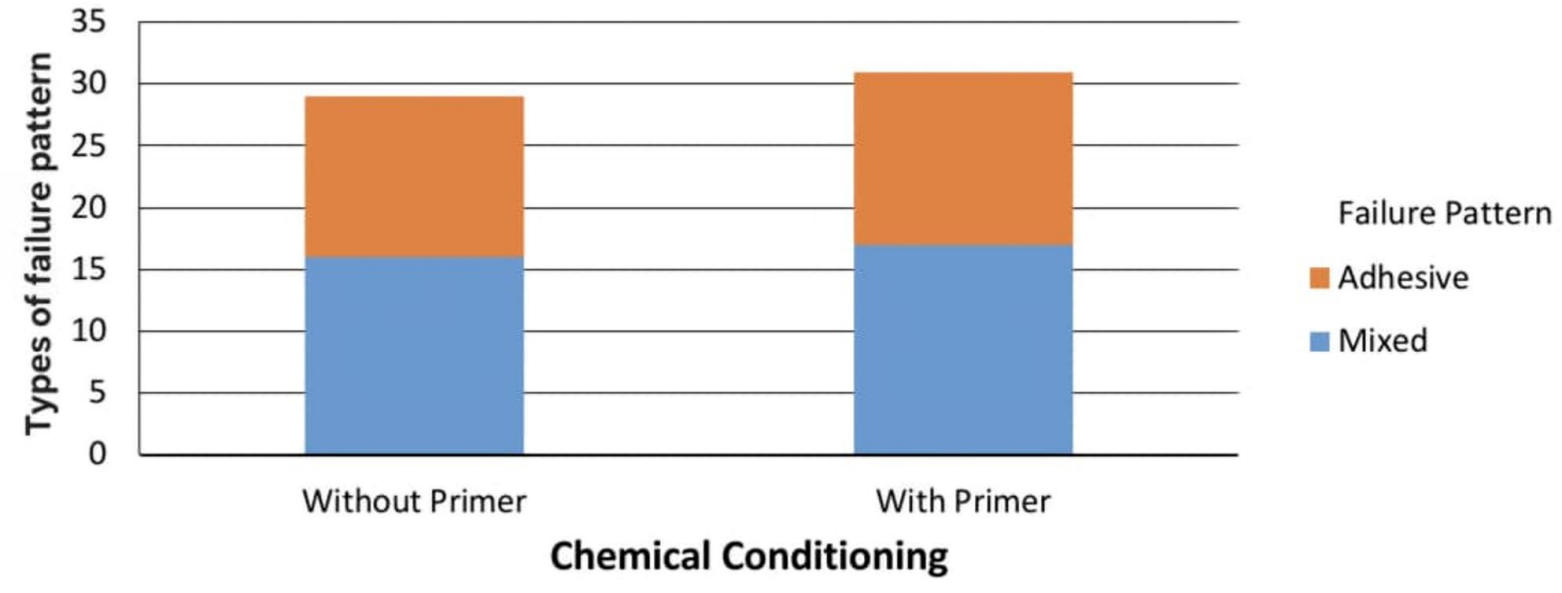
Bar-chart of failure pattern considering chemical conditioning

During aging regime two specimens were debonded from group CNT-NP, one specimen form group ABF-NP and group ZES-NP. Failure patterns of the de-bonded specimens showed 27 adhesive failure patterns and 33 mixed failure patterns with no cohesive failure. Bar-charts in (Figs. 2, 3 and 4) demonstrated failure pattern for all test groups and for each factor.

### Scanning electron microscope examination

**Fig. 5:**
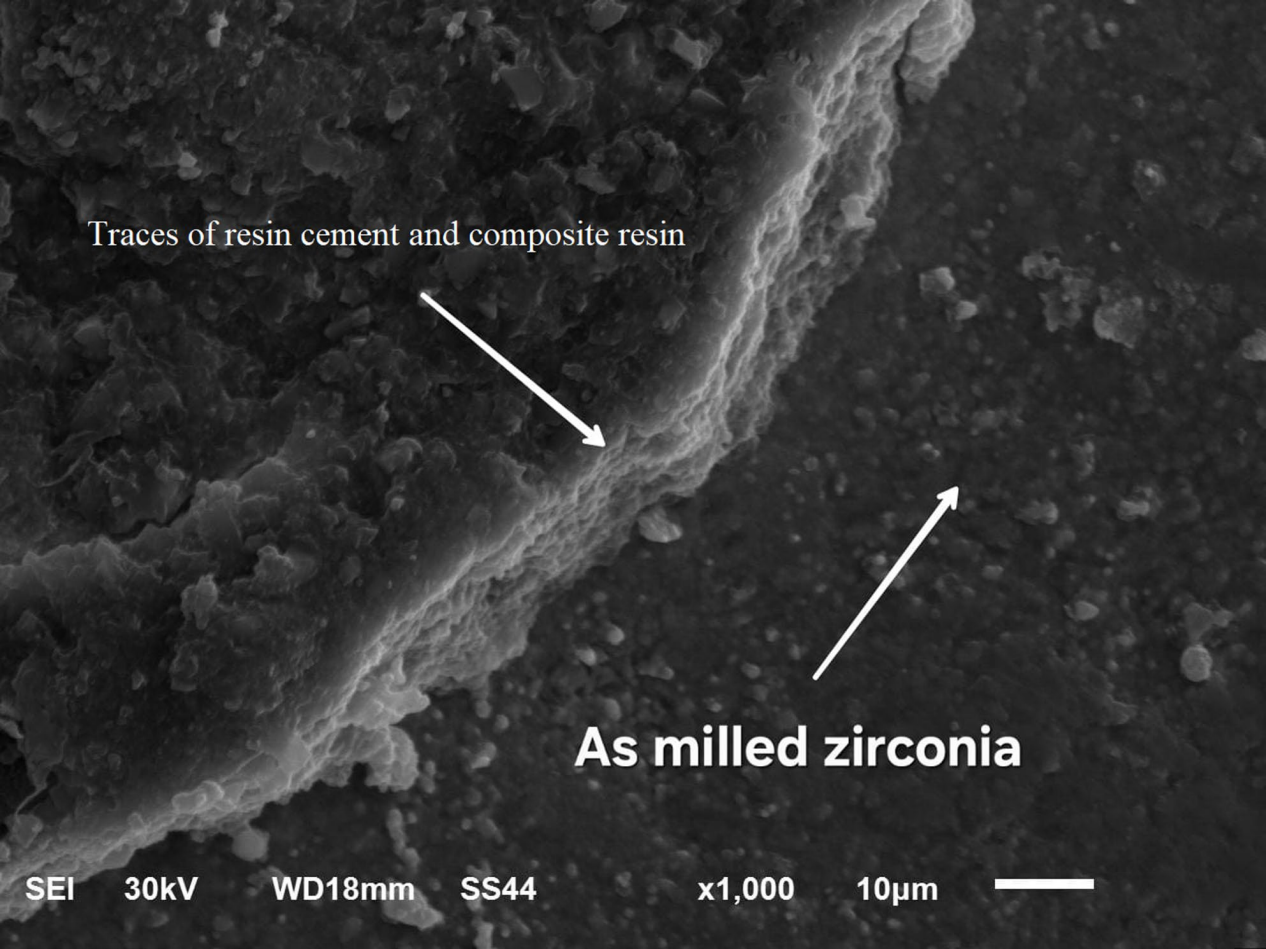
SEM micrographs at x1000 magnification of as milled specimen; Showing mixed failure mode and smooth zirconia surface with minimal irregularities

**Fig. 6:**
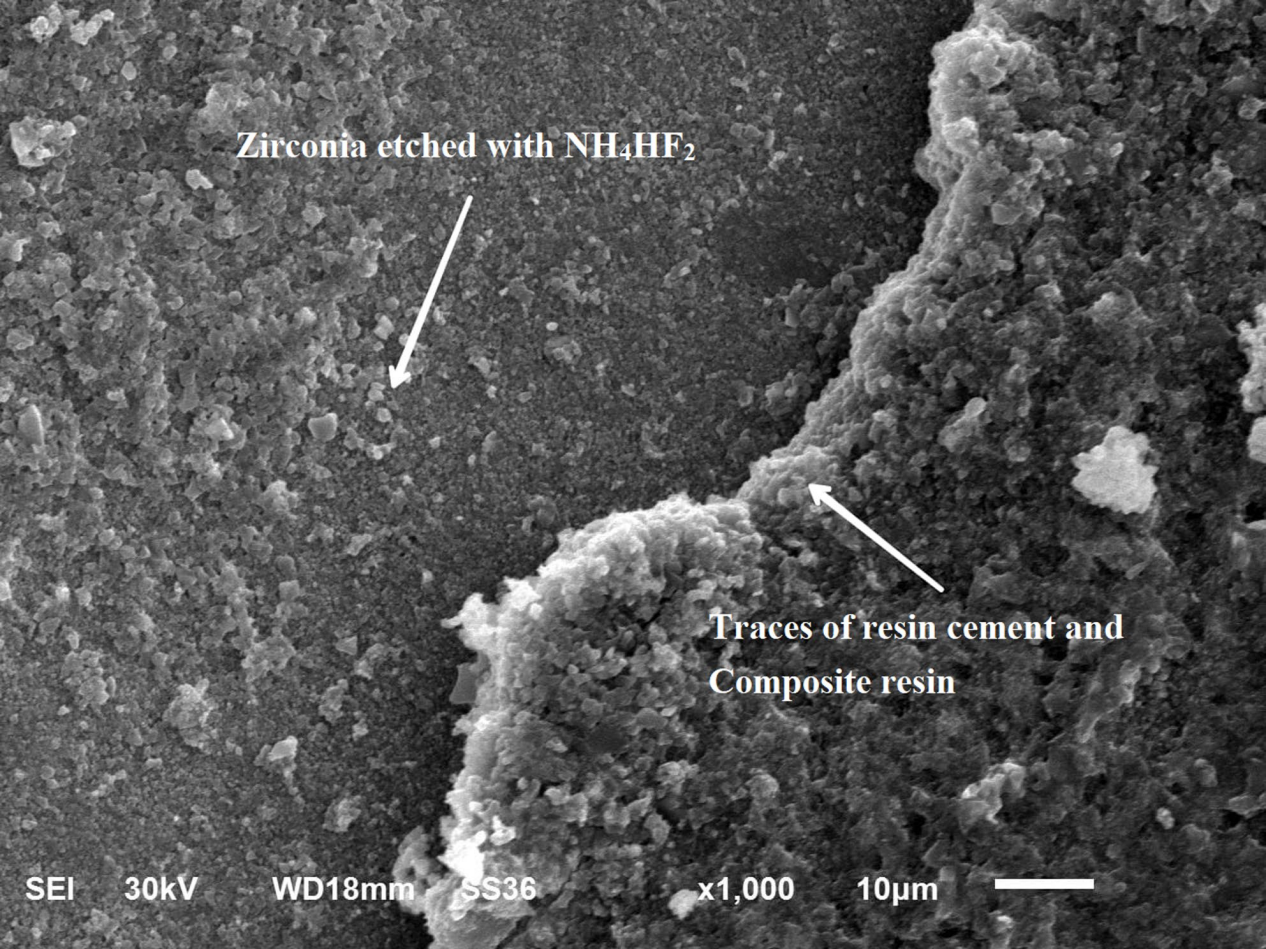
SEM micrographs at x1000 magnification of specimen treated by NH_4_HF_2_; Showing mixed failure and zirconia surface topography with unclear roughness

**Fig. 7:**
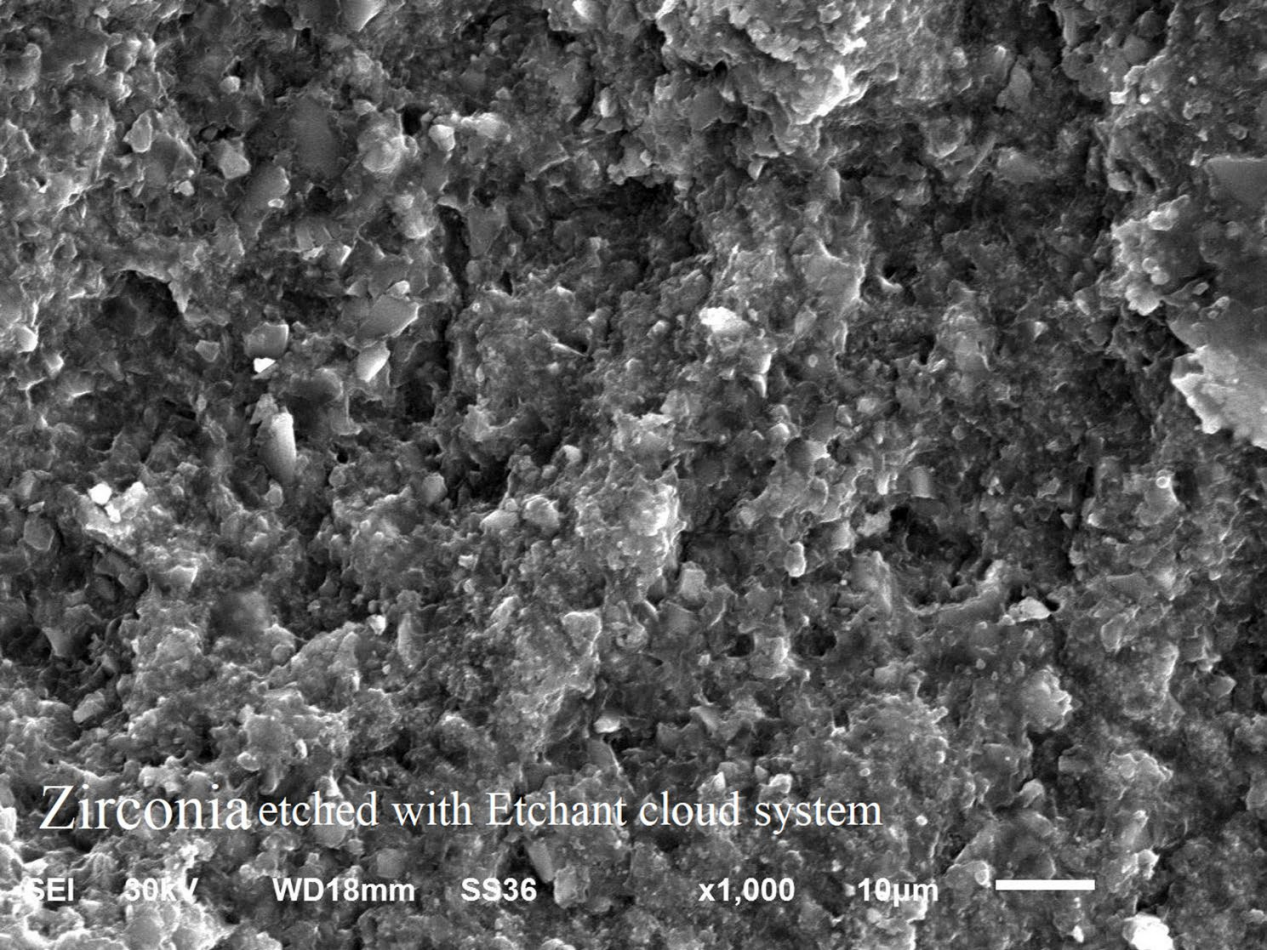
SEM micrographs at x1000 magnification of specimen treated by etchant cloud system; Showing adhesive failure and zirconia surface micro-pores with clearly defined irregularities

**Fig. 8:**
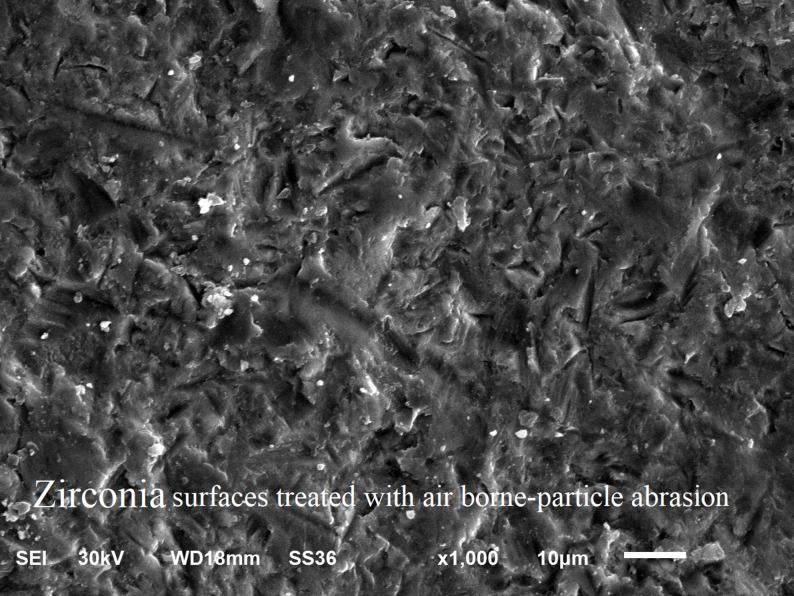
SEM micrographs at x1000 magnification of specimen treated with airborne-particle abrasion; Showing adhesive failure with pronounced surface roughness of zirconia

SEM images at X 1000 magnification showed: mixed failure mode of as milled specimen (Fig. 5), mixed mode of failure of specimen treated by NH_4_HF_2_ (Fig. 6), adhesive mode of failure of specimen treated by etchant cloud system (Fig. 7), and adhesive mode of failure of specimen treated by airborne-particle abrasion (Fig. 8).

## Discussion

The null hypothesis of the study was that micromechanical surface treatment and chemical conditioning would not have effect on the tensile bond strength (TBS) to zirconia. Results of the present study rejected the hypothesis partially. Because TBS results were significantly affected with the primer application but micromechanical surface treatment did not show significant effect on TBS. However, means TBS of all test groups ranged from (3.8-7) MPa were lower than the clinically acceptable bonding range (10-13 MPa) [4, 5]. Restorations serving in the oral cavity are subjected to thermal and mechanical stresses which can compromise bonding durability between zirconia restoration and resin cement [29]. Gale and Darvell (1999) [31] stated that 10,000 thermal cycles can simulate one year of service in the oral cavity. In this study, test groups were subjected to two artificial aging factors including long term water storage for 5 months followed by thermalcycling for 5000 cycles to simulate one year clinically.

Airborne-particle abrasion of zirconia was proved to produce surface roughness, increased surface area for bonding and increased surface energy which facilitates the flow of resin cement into intaglio surface micro-pores to form resin-ceramic micromechanical interlocking [4, 14, 28]. It could also produce hydroxyl groups on the surfaces of zirconia, which might make it more reactive with phosphate monomers [22]. Kim et al. (2021) [10] stated that zirconia surfaces that were subjected to airborne-particle abrasion with 110 μm particles obtained a surface topography with greater change making the surface optimal for a favorable bonding with less surface damage. Moreover it was reported that airborne particle-abrasion is a powerful method for strengthening zirconia ceramic by pre-stressing its surface.

Airborne-particle abrasion of intaglio zirconia surface with 110 μm Al_2_O_3_ particles was conducted in this study and was confirmed via SEM to create well defined surface roughness. This roughened surface topography was expected to enhance micromechanical interlocking and, consequently, bond strength. However, group APA-NP (4.2 MPa) did not exhibit a significant difference from the control group CNT-NP (3.4 MPa), despite the surface roughness achieved. This result might be attributed to residual alumina particles left on the bonding surfaces after airborne-particle abrasion, which could negatively impact bond strength even after ultrasonic cleaning [8]. On the other hand several literatures reported a negative effect of airborne-particle abrasion on the surface integrity of zirconia ceramic through phase transformation where tetragonal phase transformed to monoclinic phase, thus decreasing its mechanical properties [4, 10, 13]. Therefore other methods were introduced to eliminate the use of airborne-particle abrasion and omitting its negative effect on mechanical properties of zirconia restorations.

Therefore etching of zirconia using different chemicals such as HF acid and NH_4_HF_2_ was investigated in other studies [16, 18–20]. However the use of high-concentration HF acid at elevated temperatures could have risks due to potential exposure to HF vapor. Contrary, the 9% HF gel is relatively safer as the gel can be applied in small quantities to targeted areas, reducing health hazards associated with immersion in concentrated HF solutions. Furthermore, the etchant cloud system that is recently introduced to etch zirconia surface offers a triple-locking design of the safe shell along with application of the neutralizing gel to the top of shell which effectively inhibited the rapid release of HF vapor [16]. 

Hydrofluoric acid ability to etch zirconia surface was investigated in several previous studies [16, 18]. In this study, 9% HF gel was used in hot etching step of zirconia in the form of Etchant cloud system. SEM for the specimens etched by HF acid showed zirconia surface micro-retentions with clearly defined irregularities, suggesting effective acid interaction and supporting results of Kim et al. [16] who clarified that HF etching creates uniform micro-retentions without compromising zirconia’s mechanical integrity. This surface topography improved TBS to zirconia with significant difference between groups ZES-NP (6.3 MPa) and CNT-NP (3.4 MPa), (*P* = 0.026).

Zirconia etching with NH_4_HF_2_ was proved to enhance its bonding strength to resin cements in previous studies [19, 20]. Ruyter et al. (2017) [19] used NH_4_HF_2_ as powder or an aqueous slurry to etch zirconia surface with no statistically significant difference in the bond strength values. In the current study, aqueous slurries of NH_4_HF_2_ had been applied on the bonding surface of zirconia which was further heated for 10 min at 170 °C. SEM examination of the specimens etched by NH_4_HF_2_ showed surface topography with unclear roughness in comparison to air abraded surfaces or the surfaces etched by HF acid, with finer surface irregularities and some porosity. This etching process produced improved TBS in group ABF-NP (5.8 MPa) with no statistically significant difference from other test groups; ZES-NP (6.3 MPa), APA-NP (4.2 MPa) and CNT-NP (3.4 MPa).

Several literatures stated that primer application resulted in supreme bond strength between resin cements and zirconia ceramics [4, 5, 23, 24, 26]. Therefore in this study universal primer was applied to intaglio surface of zirconia to ensure chemical bonding. In agreement with other studies [4, 5, 23, 24, 26, 32–35]. primer application significantly increased TBS results. As the manufacturer reported, the universal primer contains an alcohol solution of 3-methacryloxyprophyl-trimethoxysilane (MPS) along with phosphoric acid methacrylate and sulphide methacrylate. Phosphoric acid methacrylate and sulphide methacrylate were likely added to enhance chemical bonding to oxide ceramics that contain minimal or no silica content [4, 5, 8]. 

Groups APA-P, ABF-P, and ZES-NP showed significantly higher bond strength compared to CNT-NP but did not differ significantly from group CNT-P, where only primer was applied with no previous micromechanical surface treatment. This demonstrated the enhancing effect of primer application on bond strength through chemical bonding regardless of micromechanical surface treatment. Primer application after etching with etchant cloud system, mean TBS of group ZES-P (6 MPa) was in the same range of group ZES-NP (6.3 MPa), but it was higher than mean TBS of group CNT-NP (3.4 MPa). This outcome was unexpected, as the application of primer in other groups had significantly improved bond strength. This could be attributed to the fact that chemical etching might had removed zirconia’s surface hydroxyl groups, thereby deteriorate bonding with MDP-containing resin cement [21]. 

The mixed failure pattern which was observed to be dominant in test group CNT-P after chemical conditioning with primer application indicates stronger bonding due to the effect of chemical bonding. Mixed failures suggest enhanced bonding at the resin-zirconia interface rather than adhesive failures [22]. Study limitations include that bonded specimens were subjected to thermal stresses only after storage in water. However to mimic clinical conditions specimens should be subjected to thermal and mechanical stresses in different storage media such as artificial saliva. Also, future studies should investigate effect of different conditioning techniques on bonding to new zirconia generations such as ultra, super, high translucent and mutilayer zirconia.

## Conclusions

The following conclusions were found within the limitations of this in-vitro study;

1. Zirconia surfaces treated by airborne-particle abrasion or hydrofluoric acid exhibited roughened and irregular morphologies under SEM observation, which may have contributed to improved bonding performance compared with untreated surfaces.
2. Chemical conditioning with primer application has a significant effect on the bond strength of resin cement to zirconia ceramic regardless of micro-mechanical surface treatment.
3. Combining micromechanical intaglio surface treatment with primer application would enhance clinical durability of resin bonded zirconia restorations.

## Data Availability

The data sets that were utilized and/or analyzed for this investigation can be obtained from the corresponding author at any reasonable request.

## Abbreviations

TBS: Tensile bond strength
ANOVA: Analysis of Variance
LSD: Least Significant Difference
Al_2_O_3_: Aluminum Oxide
NH4HF2: Ammonium hydrogen difluoride
HF: Hydrofluoric Acid
SPSS: Statistical Package for the Social Sciences
MPa: Megapascal
SEM: Scanning electron microscope
